# Identification of immune-related prognostic genes and construction of a risk model for Wilms tumor: A retrospective bioinformatics study

**DOI:** 10.1097/MD.0000000000049868

**Published:** 2026-07-24

**Authors:** Jin Chen, Guobin Yang, Zhihui Zhu, Jun Liao, Huajian Gu

**Affiliations:** a School of Clinical Medicine, Guizhou Medical University, Guiyang, China; c Department of Pediatric Surgery, Guizhou Medical University Affiliated Hospital, Guiyang, China; b Department of Pediatric Surgery, Guiyang Children’s Hospital, Guiyang, China

**Keywords:** drug sensitivity, prognostic markers, survival analysis, tumor microenvironment, Wilms tumor

## Abstract

Wilms tumor (WT) is the most common pediatric renal malignancy. Reliable prognostic markers are crucial for improving patient outcomes. Immune-related genes (IRGs) significantly influence tumor progression and the tumor microenvironment, yet their prognostic value in WT remains unclear. This study aimed to develop an immune-related prognostic model for WT and investigate its underlying molecular and immunological mechanisms. We analyzed RNA-seq data and clinical information from the TARGET-WT and Gene Expression Omnibus databases. Using differential expression analysis, we identified differentially expressed genes. We identified immune-related differentially expressed genes (DEIRGs) by intersecting differentially expressed genes with known IRGs. Using univariate and multivariate Cox regression along with Least Absolute Shrinkage and Selection Operator regression, we selected 4 DEIRGs and constructed a prognostic risk score model. We further analyzed the model’s molecular and immunological characteristics. Four DEIRGs (epidermal growth factor [EGF], teratocarcinoma-derived growth factor 1 [TDGF1], leukotriene B4 receptor [LTB4R], and HLA-DMB) showed significant associations with overall survival in WT patients. The risk stratification model categorized patients into high- and low-risk groups, with significantly poorer survival in the high-risk group (*P* < .001). Enrichment analysis revealed that the high-risk group showed enrichment in oncogenic pathways (e.g., genome instability), whereas the low-risk group demonstrated enrichment in immune defense and homeostasis pathways. The high-risk group exhibited reduced tumor microenvironment (TME) immunoreactivity, with EGF and LTB4R emerging as key regulatory factors. Both EGF and LTB4R demonstrate differential expression across multiple tumor types and correlate significantly with TME scores. The immune-related prognostic model developed in this study elucidates the regulatory roles of EGF and LTB4R in Wilms tumor progression. This model effectively stratifies patients, enables accurate prognosis prediction, facilitates individualized treatment planning, and identifies potential therapeutic targets.

## 1. Introduction

Wilms tumor (WT) represents the most prevalent pediatric renal malignancy. Accounting for approximately 6% to 7% of all pediatric solid tumors,^[1]^ While multimodal therapy (surgery with chemoradiation) achieves >90% overall survival, high-risk patients frequently experience poor outcomes due to recurrence or drug resistance^[2,3]^ Current prognostic evaluation primarily relies on histopathological classification and staging systems (e.g., Children’s Oncology Group [COG]/International Society of Paediatric Oncology [SIOP] criteria).^[4]^ However, these parameters incompletely capture tumor molecular heterogeneity and inadequately predict responses to individualized therapies. Emerging research highlights the tumor microenvironment’s (TME) crucial role in cancer progression and therapeutic response. Research demonstrates that dysregulated immune-related gene (IRG) expression modulates tumor immune evasion and chemosensitivity through T-cell infiltration and cytokine secretion.^[5]^ However, comprehensive IRG profiling in WT and its prognostic significance remain underexplored. This knowledge gap impedes development of precision immunotherapies targeting the tumor microenvironment.

Current molecular studies of WT primarily investigate driver mutations (e.g., WT1, TP53, CTNNB1)^[6]^ and epigenetic alterations (e.g., loss of imprinting, miRNA dysregulation).^[7,8]^ While certain genes (e.g., SIX2, NCAM1) show prognostic associations,^[9,10]^ their mechanisms remain largely confined to tumor cell proliferation/differentiation, without comprehensive immune microenvironment analysis. Recent adult solid tumor studies demonstrate that immune-related prognostic models (e.g., TIDE score, ESTIMATE algorithm) can predict survival and treatment response by integrating IRG expression profiles with TME characteristics.^[11-14]^ However, these models remain underutilized in pediatric tumors. Furthermore, while some studies suggest WT (nephroblastoma) has low immunogenicity and limited therapeutic potential, single-cell sequencing reveals heterogeneous immune cell infiltration (e.g., exhausted T cells, M2 macrophages) in the TME, indicating potentially underestimated immune regulation.^[15]^

The primary objective of this study was to identify key immune prognostic genes in WT and develop a clinically applicable risk prediction model. The secondary objectives were to: investigate the molecular and immunological mechanisms underlying the risk model, explore the role of hub genes (epidermal growth factor [EGF] and leukotriene B4 receptor [LTB4R]) in the tumor immune microenvironment, and identify potential therapeutic implications. This research not only elucidates the molecular mechanisms of immunoregulation in Wilms’ tumor, but also provides a theoretical basis for early identification of high-risk patients, treatment optimization, and discovery of new immune targets. This study has significant scientific value and clinical potential.

## 2. Materials and method

### 2.1. Data collection

From The Cancer Genome Atlas (TCGA) database (https://portal.gdc.cancer.gov/), we obtained RNA-seq data from 130 TARGET-WT samples, 6 adjacent normal samples, and corresponding clinicopathological data. Samples lacking OS data were excluded. We also analyzed mRNA expression profiles from 33 cancer types and extracted model feature genes. Additionally, we downloaded RNA-seq datasets (GSE66405, GSE11151, GSE11024, and GSE73209) and clinical data for WT from the Gene Expression Omnibus (GEO) database (https://www.ncbi.nlm.nih.gov/geo/).^[16]^ IRG sets were obtained from the Immunology Database and Analysis Portal (ImmPort).

### 2.2. Selection bias and control measures

We acknowledge several potential selection biases in our retrospective bioinformatics study design. First, reliance on public databases introduces potential selection bias from nonrandom patient enrollment and variable data quality across institutions. Second, limited sample sizes in some GEO datasets may affect statistical power and generalizability. Third, batch effects between different sequencing platforms and time periods could introduce technical variation. Fourth, the inclusion criteria requiring complete OS data may create survival bias.

To minimize these biases, we implemented several measures: used multiple independent datasets (TARGET-WT, GSE66405, GSE11151, GSE11024, GSE73209) for discovery and exploratory evaluation, acknowledging that true external validation was not performed in this retrospective study, applied stringent and uniform statistical thresholds (|log_2_FC| > 0.585, adjusted *P* < .05) across all analyses, standardized data processing pipelines using consistent R packages and versions, employed robust statistical methods including Least Absolute Shrinkage and Selection Operator (LASSO) regression to prevent overfitting, performed multivariate Cox regression incorporating the 4 identified prognostic genes to construct the risk model, and assessed model stability through internal validation using the same cohort.

### 2.3. Ethical approval statement

This study utilized publicly available de-identified data from TCGA and GEO databases. No human subjects were involved, and no personal identifiers were accessed. Therefore, institutional review board approval was not required for this study.

### 2.4. Differential gene expression analysis and screening

We performed differential expression analysis using the R package “limma^[17]^” on expression matrices from the GSE66405 and GSE11151 datasets. We identified differentially expressed genes (DEGs) between tumor and adjacent normal tissues using thresholds of |log_2_FC| > 0.585 and adjusted *P*-value < .05. From the Immunology Database and Analysis Portal (ImmPort; (http://www.immport.org), We downloaded IRGs and intersected them with DEGs from both datasets to identify differentially expressed immune-related genes (DEIRGs).

### 2.5. Prognostic analysis

We obtained OS data for TCGA tumor samples. Using the “survival” R package, we performed Cox proportional hazards analysis to assess associations between gene expression levels and patient survival. We generated forest plots using the “forestplot” R package. We conducted survival analysis using the Kaplan–Meier method with log-rank tests (significance threshold *P* < .05). We plotted survival curves using the “survminer” and “survival” R packages.

### 2.6. Construction of prognostic risk score model

Using RNA-seq and survival data from 130 patients in the TARGET-WT database, We analyzed 4 differentially expressed immune-related genes (DEIRGs: EGF, teratocarcinoma-derived growth factor 1 [TDGF1], LTB4R, and Major Histocompatibility Complex Class II DM Beta [HLA-DMB]) using LASSO-Cox regression with the “glmnet” R package. We constructed a polygenic risk score model. The risk score was calculated as: RiskScore = 0.123 × Expression of EGF + −0.088 × Expression of TDGF1 + 0.356 × Expression of LTB4R + −0.057 × Expressionof HLA-DMB. Patients were stratified into high-risk (median score above cutoff) and low-risk (median score below cutoff) groups. We compared OS between groups using Kaplan–Meier analysis and generated time-dependent receiver operating characteristic (ROC) curves with the “timeROC” R package. We calculated the area under the curve to evaluate model accuracy and predictive performance.

### 2.7. Enrichment analysis

To investigate the molecular mechanisms of DEIRGs, Using the “clusterProfiler” R package, we performed Gene Ontology (GO) and Kyoto Encyclopedia of Genes and Genomes (KEGG) enrichment analyses with a significance threshold of *P* < .05. GO analysis included 3 categories: biological processes (BPs), molecular functions, and cellular components. We visualized enrichment results using the “ggplot2” R package. Additionally, we obtained gene sets from the Molecular Signatures Database (MSigDB; https://www.gsea-msigdb.org) and performed gene set variation analysis (GSVA) using the “GSVA” R package. GSVA scores were calculated for each sample. Using the “limma” R package, we compared GSVA scores between high- and low-risk groups, identifying significantly different pathways (*P* < .05). We constructed protein–protein interaction networks using the STRING database (medium confidence score: 0.4). Interaction networks were visualized using Cytoscape software.

### 2.8. Drug sensitivity prediction

We performed drug sensitivity analysis using the “pRRophetic^[18]^” R package. This package predicts drug response from gene expression data and calculates IC50 values (half-maximal inhibitory concentration), which indicates drug potency for inducing apoptosis. Lower IC50 values indicate greater drug potency. We compared IC50 values between high-risk and low-risk groups and analyzed sensitivity differences using Wilcoxon rank-sum tests (*P* < .05) to identify potential therapeutic agents.

### 2.9. Evaluation of tumor iImmune microenvironment

We analyzed TARGET-WT data using the “ESTIMATE^[19]^” R package to calculate immune scores, stromal scores, tumor purity, and ESTIMATE scores. This allowed assessment of the tumor immune microenvironment. Simultaneously, we employed single-sample gene set enrichment analysis to quantify infiltration levels of 28 immune cell types in TARGET-WT samples. We compared immune cell infiltration differences between high-risk and low-risk groups using Wilcoxon rank-sum tests. Additionally, we generated correlation heatmaps using the “corrplot” R package. This analysis revealed associations between immune cell infiltration, immune gene expression, and characteristic gene expression.

### 2.10. Statistical analysis

We conducted all statistical analyses and data visualizations using R software (version 4.4.1; R Foundation for Statistical Computing, https://www.r-project.org). We compared mRNA expression between tumor and normal tissues using Wilcoxon signed-rank tests. We evaluated correlations between continuous variables using Spearman’s rank correlation analysis. We generated Kaplan–Meier survival curves to analyze survival differences, with statistical significance determined by log-rank tests. A *P*-value threshold of <.05 was used to determine statistical significance.

## 3. Result

### 3.1. Identification of DEGs and functional enrichment analysis

**Figure 1.**
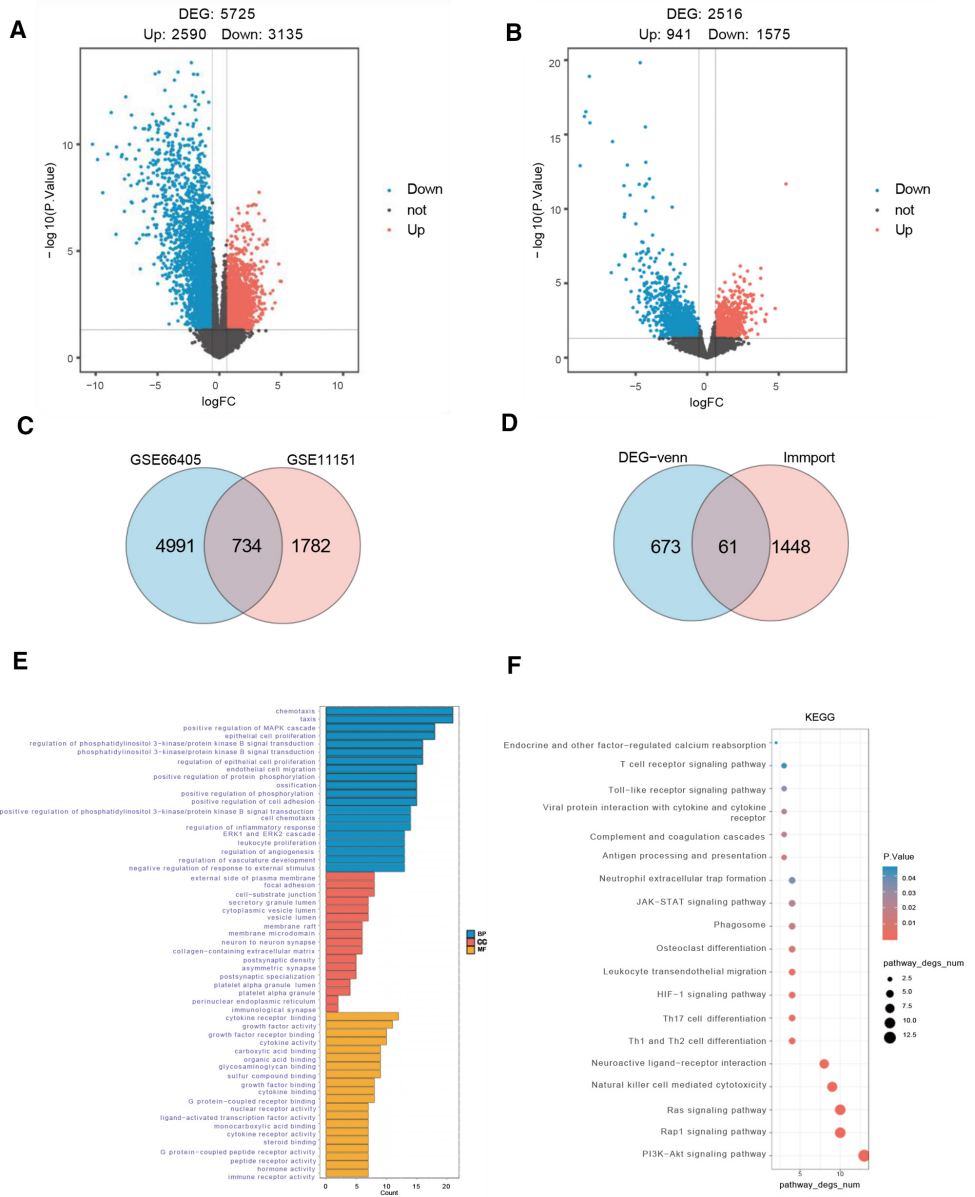
Identification and functional analysis of differentially expressed genes (DEGs) in Wilms’ tumor. (A) Volcano plot of DEGs between Wilms’ tumor and normal tissues in dataset GSE66405 (adjusted *P* < .05, |log_2_FC| > 0.585). Red and blue dots represent significantly up- and downregulated genes in tumor tissues, respectively. (B) Volcano plot of DEGs in validation dataset GSE11151 (same thresholds). (C) Venn diagram showing overlapping DEGs between GSE66405 and GSE11151. (D) Venn diagram of shared DEGs overlapping with immune-related genes. (E) Gene Ontology (GO) enrichment analysis of common DEGs (top 20 significant biological processes shown; *P* < .05). (F) KEGG pathway enrichment analysis of common DEGs (top 20 significant pathways shown; *P* < .05). DEG = differentially expressed gene, GO = Gene Ontology, KEGG = Kyoto Encyclopedia of Genes and Genomes.

Analysis of RNA-seq data from GSE66405 and GSE11151 identified 5725 and 2516 DEGs, respectively. The GSE66405 dataset contained 2590 upregulated and 3135 downregulated genes (Fig. 1A), while GSE11151 contained 941 upregulated and 1575 downregulated genes (Fig. 1B). Intersection analysis revealed 734 common DEGs between the 2 datasets (Fig. 1C). Further intersection with IRGs identified 61 DEIRGs (Fig. 1D).

To investigate the functional roles of these DEIRGs, we conducted GO and KEGG enrichment analyses. GO analysis revealed significant enrichment in: cell chemotaxis, spatial organization of intracellular vesicles and secretory granules, and growth factor/cytokine binding-related BPs including membrane fusion and signal transduction (Fig. 1E). KEGG pathway analysis demonstrated significant involvement in: phosphatidylinositol 3-kinase (PI3K)-Akt signaling, Rap1 signaling, and Ras signaling pathways (Fig. 1F), all of which play crucial roles in cellular signal transduction. Protein–protein interaction network analysis identified 39 core DEIRGs with extensive interaction patterns (Fig. S1, Supplemental Digital Content 1). These core genes likely regulate key BPs.

### 3.2. Identification of prognostic key genes in WT patients and construction and development of a risk scoring model

**Figure 2.**
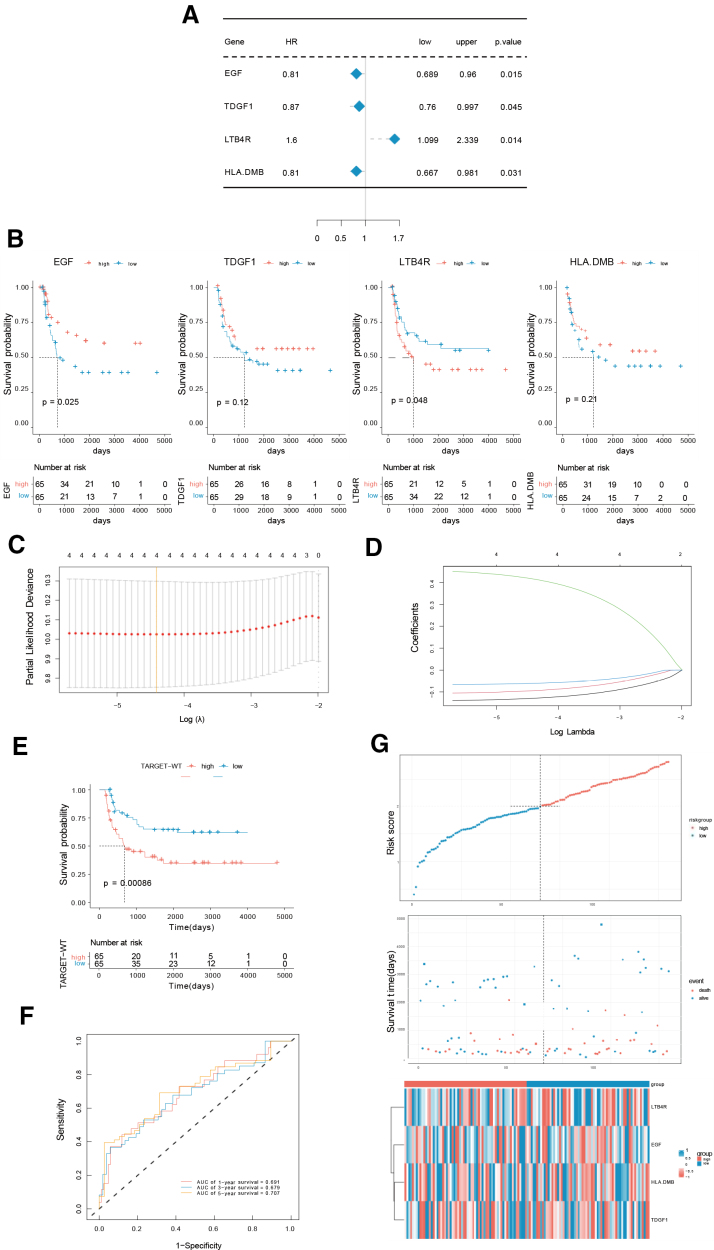
Prognostic model construction and validation based on DEIRGs in the TCGA-WT cohort. (A) Forest plot for univariate Cox analysis (*P* < .05). (B) Kaplan–Meier survival curves of 4 prognostic DEIRGs (log-rank test *P* < .05). (C) LASSO regression analysis plot for 4 prognostic DEIRGs (optimal lambda selected at the red dotted line where the partial likelihood deviance is minimized). (D) Bar plot of multivariate Cox regression analysis coefficients for feature genes (*y*-axis: coefficient values; bottom *x*-axis: log(λ); top *x*-axis: number of nonzero coefficients). (E) KM survival analysis of risk groups in the TCGA training set (blue: Low_Risk; red: High_Risk; log-rank *P* < .001). (F) Time-dependent ROC curves for 1-, 3-, and 5-year survival predictions using the prognostic model (AUC values shown). (G) Distribution of risk scores and survival times for each patient sample in the training set (risk score distribution (top), survival status (middle), and signature gene expression heatmap (bottom) for TCGA patients). AUC = area under the curve, DEIRG = differentially expressed immune-related gene, KM = Kaplan-Meier, LASSO = Least Absolute Shrinkage and Selection Operator, ROC = receiver operating characteristic, TCGA = The Cancer Genome Atlas.

To identify prognostic biomarkers in WT, we analyzed expression data for 61 DEIRGs from the TARGET-WT dataset. Univariate Cox regression analysis identified 4 DEIRGs (EGF, TDGF1, LTB4R, and HLA-DMB) significantly associated with OS (*P* < .05, Fig. 2A). Kaplan–Meier analysis confirmed that expression levels of these 4 genes significantly correlated with patient survival (*P* < .05; Fig. 2B).

We conducted LASSO and multivariate Cox regression analyses on these 4 DEIRGs (Fig. 2C, D). These analyses enabled construction of a prognostic risk scoring model using these 4 DEIRGs. Using the median risk score as cutoff, we stratified the TARGET-WT cohort into high-risk and low-risk groups. Kaplan–Meier analysis revealed significantly poorer survival in high-risk versus low-risk patients (*P* < .001; Fig. 2E). Time-dependent ROC analysis yielded area under the curve values of 0.691 (1-year), 0.679 (3-year), and 0.707 (5-year) for OS prediction (Fig. 2F), demonstrating good model performance. We ranked all patients in the TARGET-WT cohort by risk score, showing increasing risk scores from left to right. Dot plot visualization demonstrated an inverse relationship between risk scores and survival outcomes. Heatmap analysis revealed distinct expression patterns of these DEIRGs between risk groups (Fig. 2G). Our prognostic model demonstrated strong accuracy for predicting OS in WT patients.

### 3.3. Molecular characterization of prognostic risk score subgroups

**Figure 3.**
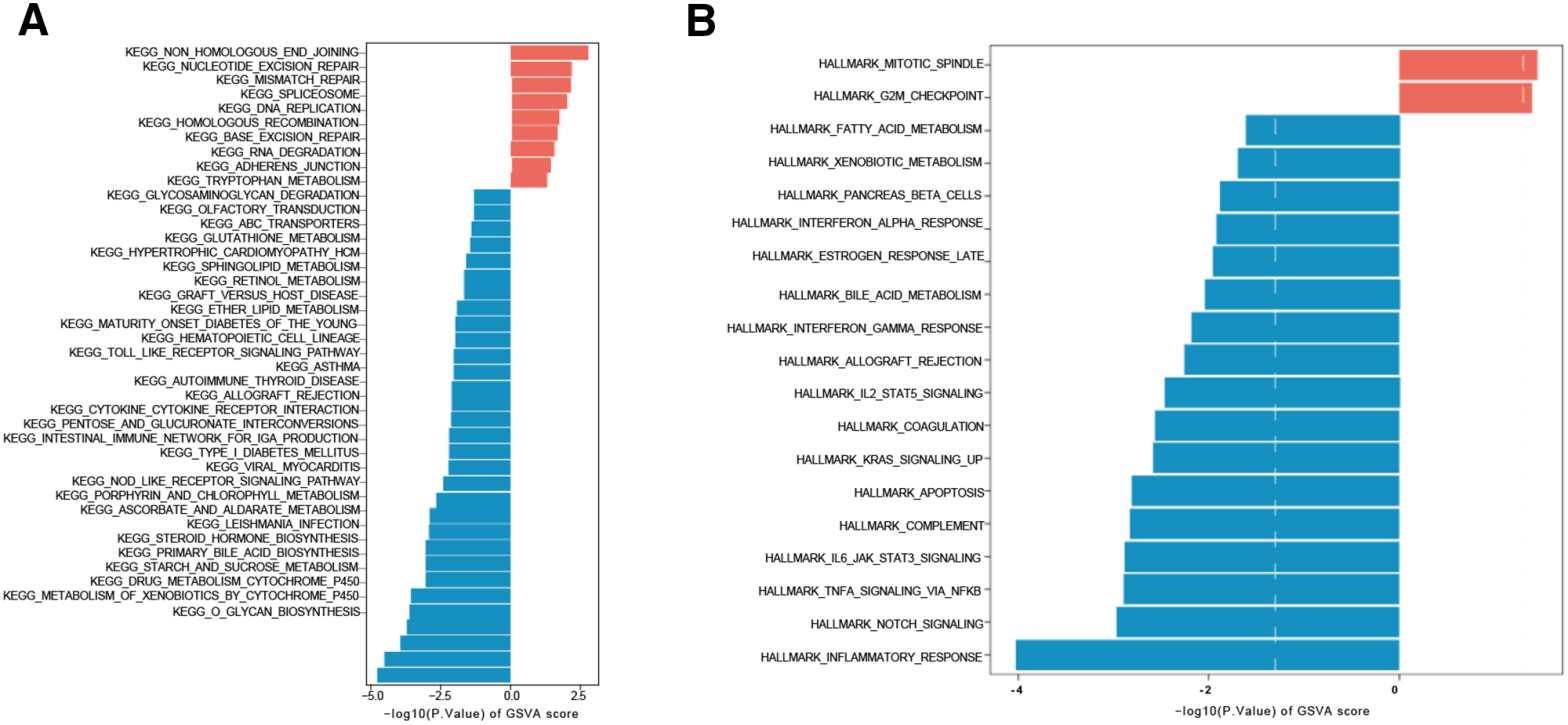
Functional analysis of prognostic risk score model feature genes. The KEGG pathway (A) and the HALLMARK gene set (B) enrichment analysis by GSVA (the significantly upregulated (red) and downregulated (blue) pathways are displayed; *P* < .05). GSVA = gene set variation analysis, HALLMARK = Molecular Signatures Database Hallmark Gene Sets, KEGG = Kyoto Encyclopedia of Genes and Genomes.

To characterize biological functions associated with our risk model genes, we conducted GSVA. KEGG pathway analysis revealed significant enrichment of cell cycle and DNA repair pathways including DNA replication, homologous recombination, mismatch repair, nucleotide excision repair, and nonhomologous end joining in high-risk patients metabolic pathways including carbohydrate, lipid, and amino acid metabolism, along with bile acid synthesis, were predominantly enriched in low-risk patients (Fig. 3A). HALLMARK gene set analysis further confirmed enrichment of cell cycle pathways, particularly mitotic spindle formation, DNA repair, and G2/M checkpoint regulation (Fig. 3B). This cell cycle enhancement in high-risk patients, consistent with their poor prognosis. Conversely, low-risk patients showed enrichment in homeostatic pathways including apoptosis, suggesting tumor suppression through maintained cellular homeostasis (Fig. 3B). Our risk model effectively distinguishes molecular subtypes in WT, with high-risk patients exhibiting pro-tumorigenic pathways and low-risk patients maintaining normal cellular functions.

### 3.4. Immunological characterization of prognostic risk model subgroups

**Figure 4.**
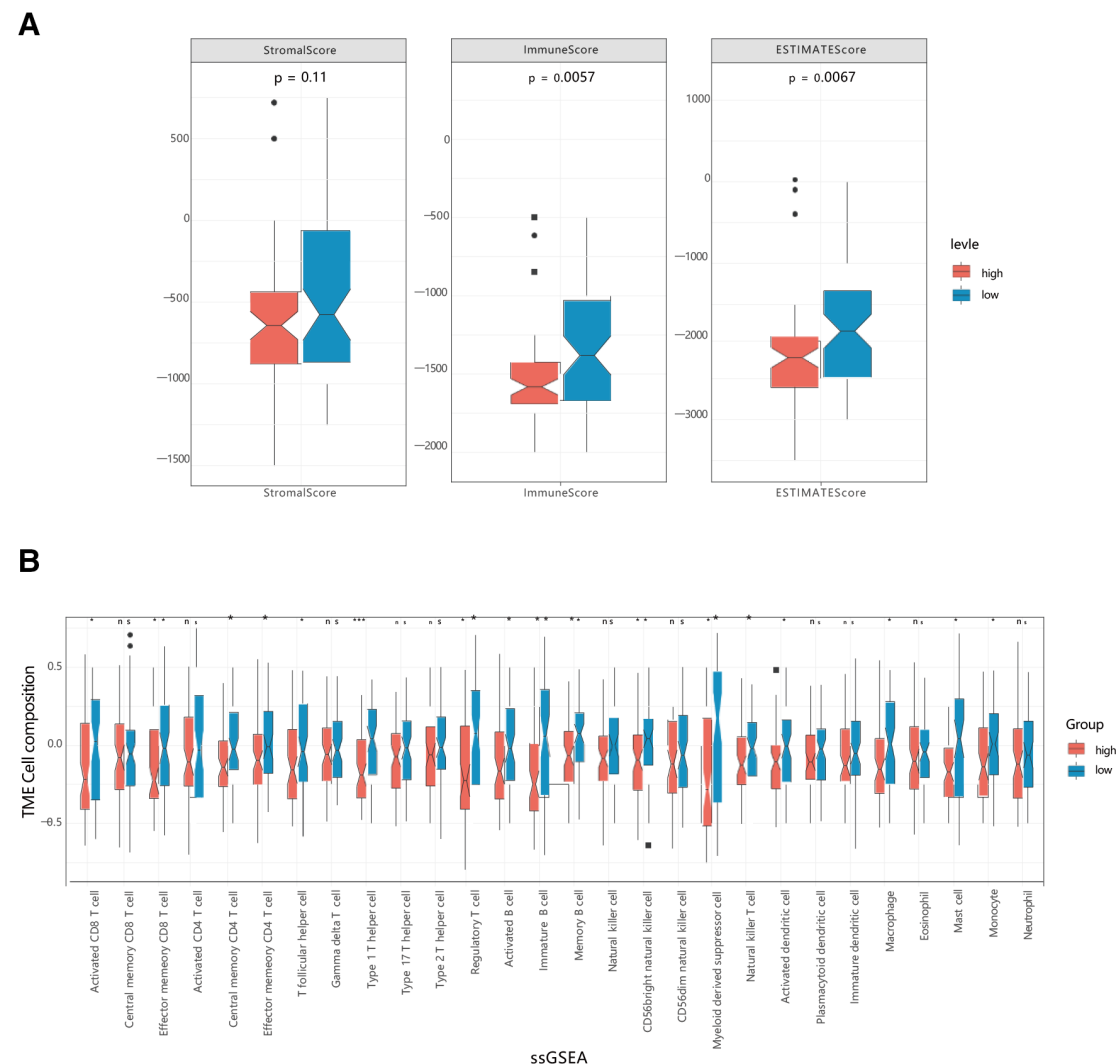
Tumor microenvironment characterization and immune cell infiltration analysis in the TARGET-WT cohort. (A) ESTIMATE algorithm-derived scores showing immune score (left), stromal score (middle), and combined ESTIMATE score (right) across tumor samples. (B) ssGSEA-quantified immune cell infiltration proportions displayed as boxplots, comparing High_Risk (red) and Low_Risk (blue) groups (Wilcoxon rank-sum test, *P < .05, **P < .01, ***P < .001). ssGSEA= single-sample gene set enrichment analysis, WT = Wilms tumor.

To characterize immunological differences between risk subgroups, we analyzed tumor microenvironment components. Significant differences emerged in immune scores and ESTIMATE scores between high-risk and low-risk subgroups (*P* < .05, Fig. 4A), reflecting distinct immune microenvironment compositions. To compare immune cell infiltration patterns between high-risk and low-risk groups, we quantified infiltration levels of 28 immune cell types using single-sample gene set enrichment analysis. Significant infiltration differences were observed for multiple immune cell types, including activated CD8+ T cells, regulatory T cells (Tregs), immature B cells, monocytes, and mast cells (*P* < .05, Fig. 4B). These differential infiltration patterns between risk groups may contribute to their distinct clinical outcomes.

### 3.5. Drug sensitivity profiling of risk subgroups

**Figure 5.**
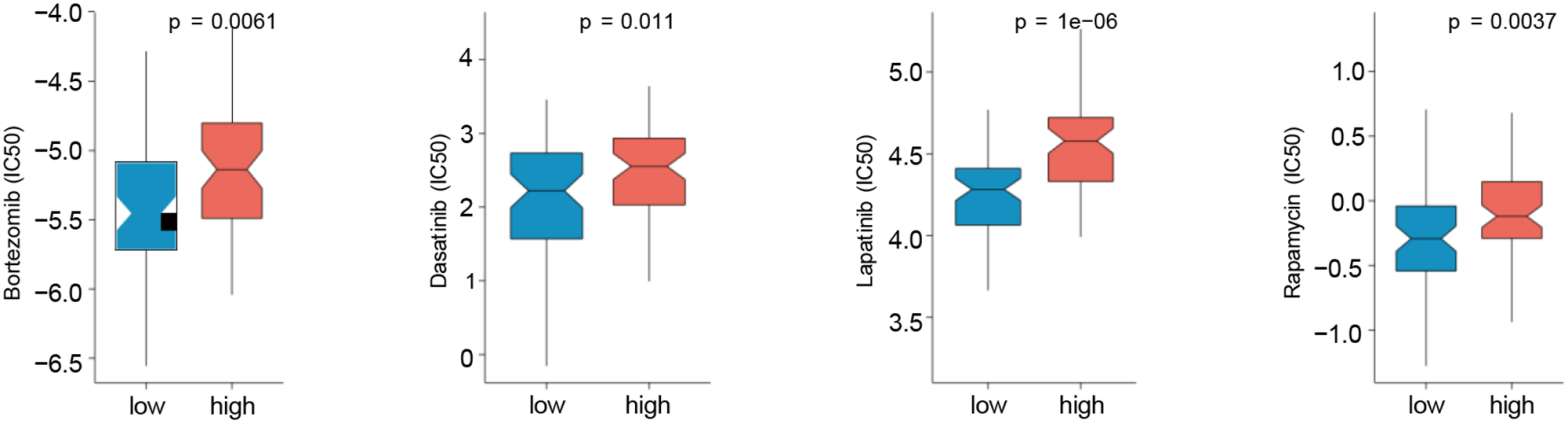
Drug sensitivity analysis between risk groups in WT patients. WT = Wilms tumor.

Using TARGET-WT data, we predicted drug sensitivity differences between high-risk and low-risk subgroups. Drug potency was evaluated via IC50, where lower values indicate greater apoptotic potential. Significantly lower IC50 values for bortezomib, dasatinib, lapatinib, and rapamycin were observed in low-risk versus high-risk subgroups, suggesting enhanced apoptotic effects and antiproliferative activity in low-risk patients (Fig. 5). Low-risk patients showed increased sensitivity to these targeted therapies. These findings may guide personalized treatment selection for WT patients.

### 3.6. Identification of hub genes in TARGET-WT

**Figure 6.**
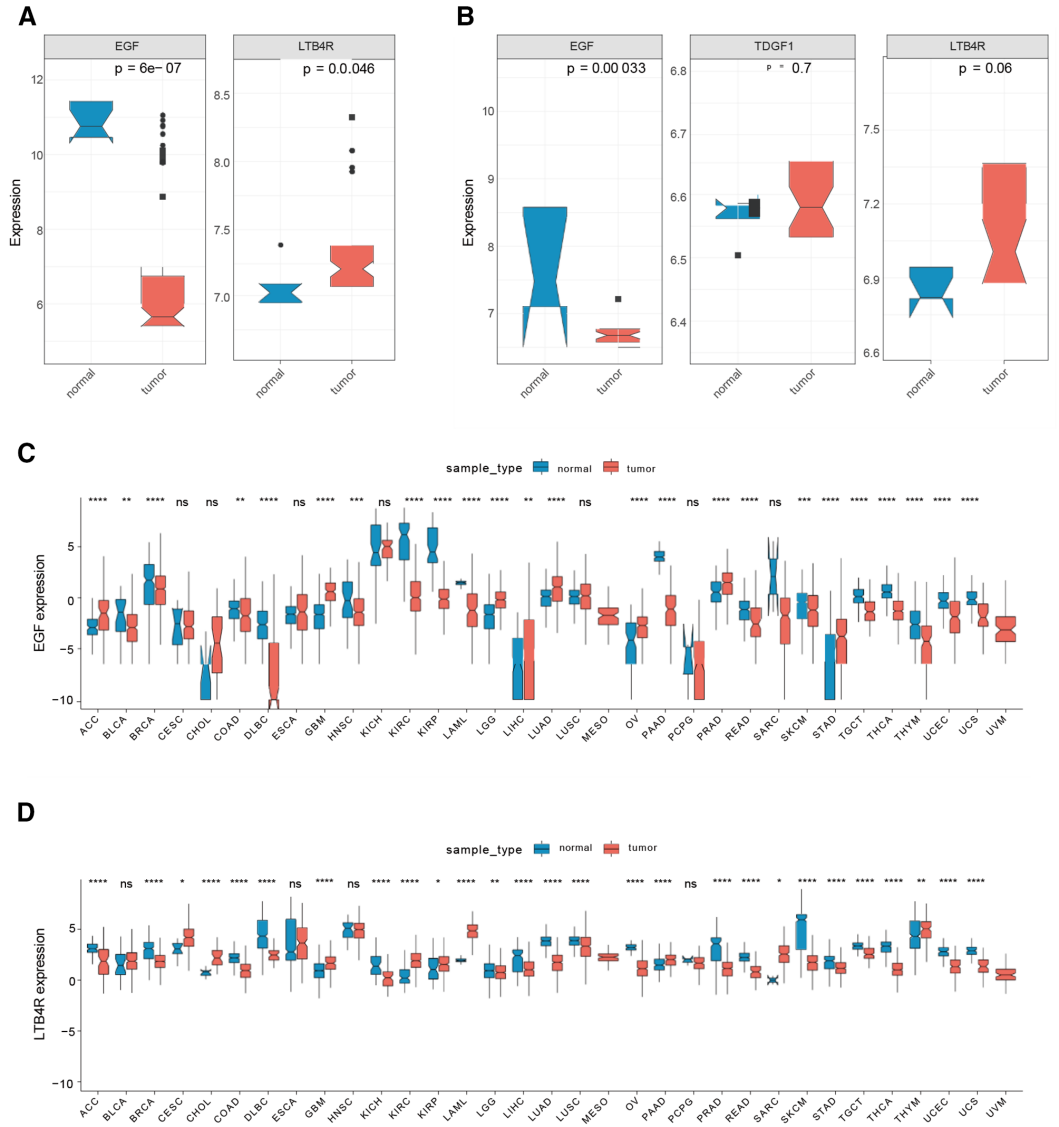
Identification and Analysis of Hub Genes in TARGET-WT. (A) Differential expression analysis of key genes (EGF, LTB4R) in GSE11024. (B) Differential expression analysis of key genes (EGF, TDGF1, LTB4R) in GSE73209. (C, D) Pan-cancer analysis of EGF (C) and LTB4R (D) expression across 33 tumor types (TCGA) versus normal tissues (GTEx). The Wilcoxon test was used to evaluate the difference between 2 subgroups (ns *P* > .05; *P < 0.05; **P < 0.01; ***P < .05;**P < .0001). EGF = epidermal growth factor, LTB4R = leukotriene B4 receptor, TCGA = The Cancer Genome Atlas, TDGF1 = teratocarcinoma-derived growth factor 1.

We analyzed hub gene expression patterns in TARGET-WT using 2 independent datasets (GSE11024 and GSE73209). EGF showed significantly lower expression across all tumor samples (*P* < .001; Fig. 6A), while LTB4R upregulation was specific to GSE11024 (*P* = .0046), with no significant differential expression in GSE73209 (*P* = .06; Fig. 6B). Based on consistent patterns, we identified EGF and LTB4R as hub genes for further investigation. We extended our analysis to multiple cancer types to validate pan-cancer expression profiles of EGF and LTB4R. EGF showed significantly lower expression in bladder urothelial carcinoma (BLCA), head and neck squamous cell carcinoma, kidney renal clear cell carcinoma, and pancreatic adenocarcinoma (*P* < .05; Fig. 6C). Conversely, LTB4R exhibited higher expression in cervical squamous cell carcinoma, cholangiocarcinoma, and glioblastoma (*P* < .05; Fig. 6D). These findings suggest EGF and LTB4R have significant roles in WT and potential pan-cancer biological functions.

### 3.7. Correlation between hub genes and tumor immune microenvironment

**Figure 7.**
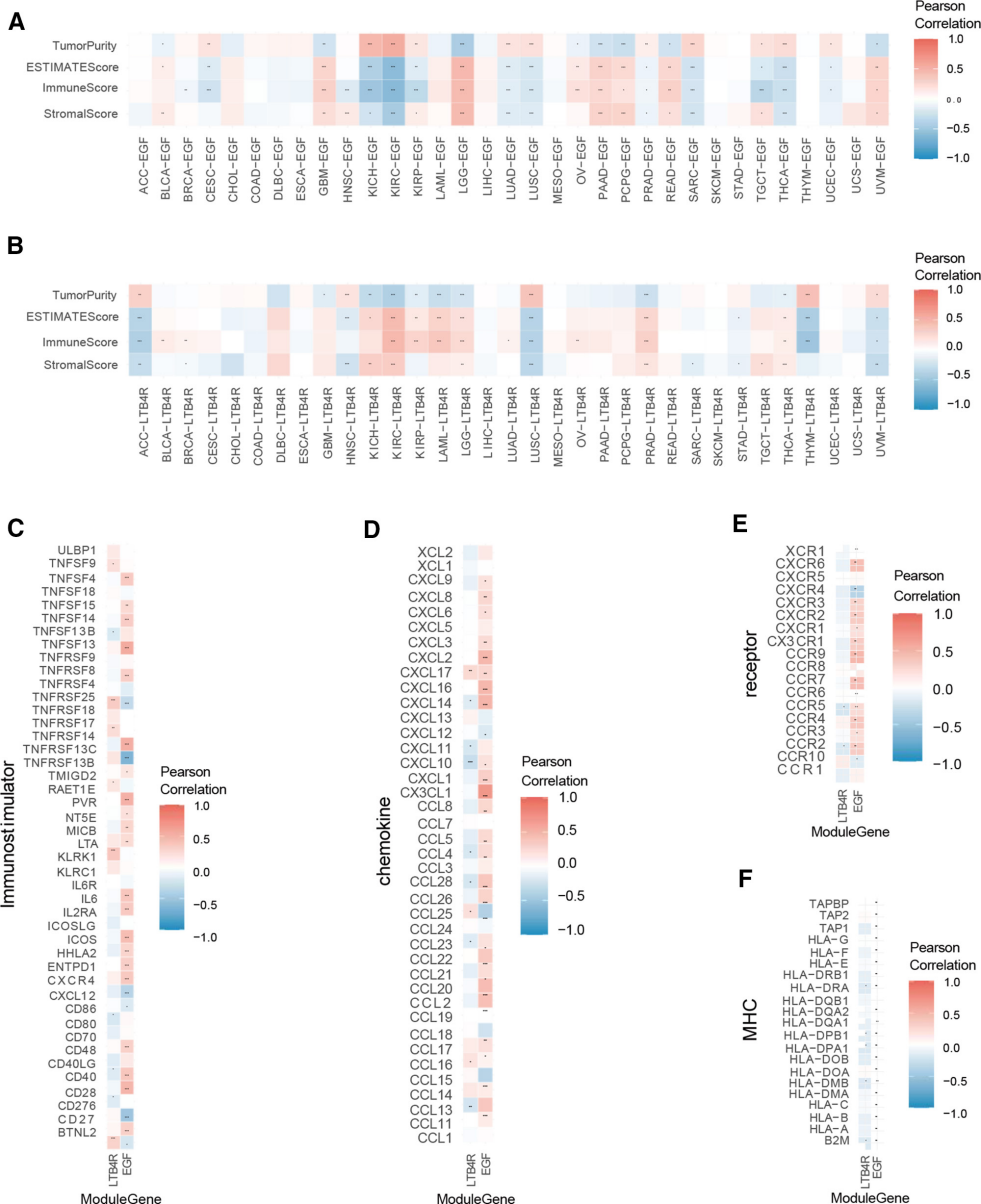
Correlation analysis between Hub gene and tumor immune microenvironment. (A, B) Heatmap showing correlation coefficients between EGF (A) and LTB4R (B) expression and ESTIMATE scores (Purity/ESTIMATE/Immune/Stromal) in pan-cancer analysis. (C–E) Immune feature association heatmaps for both genes (EGF and LTB4R) with: (C) immunostimulators, (D) chemokines, (E) chemokine receptors, and (F) MHC molecules (color scale: red = positive, blue = negative correlation; *P < .05;**P < .01;***P < .001;****P < .0001). EGF = epidermal growth factor, LTB4R = leukotriene B4 receptor, MHC = major histocompatibility complex.

To investigate associations between hub genes (EGF and LTB4R) and the tumor immune microenvironment, we employed the ESTIMATE algorithm to correlate their pan-cancer expression with tumor microenvironment scores. EGF expression showed significant negative correlations with stromal scores, immune scores, and ESTIMATE scores across multiple cancers. while showing positive correlation with tumor purity (Fig. 7A). Conversely, LTB4R exhibited inverse correlations with these microenvironment scores (Fig. 7B). In BLCA, EGF correlated positively with stromal score (*R* = 0.13, *P* = .0053) and ESTIMATE score (*R* = 0.12, *P* = .013), but negatively with tumor purity (*R* = −0.11, *P* = .026; Fig. S2A, Supplemental Digital Content 2). LTB4R demonstrated only 2 significant association in BLCA – a positive correlation with immune score (*R* = 0.13, *P* = .0077; Fig. S2B, Supplemental Digital Content 2).

We additionally examined correlations between EGF/LTB4R expression and IRGs, including immunostimulators, chemokines, chemokine receptors, and major histocompatibility complex molecules. EGF expression positively correlated with most immunostimulators, whereas LTB4R showed inverse correlations (Fig. 7C). Similar patterns emerged for chemokines, chemokine receptors, and major histocompatibility complex molecules (Fig. 7D–F).

## 4. Discussion

Biomarkers are increasingly valuable for predicting outcomes in WT management.^[20]^ Prognostic biomarkers significantly contribute to WT diagnosis and treatment. They aid disease staging, treatment response evaluation, and relapse risk prediction.^[21]^ Liquid biopsy assessment of these biomarkers may enhance therapeutic efficacy while reducing treatment-related toxicity.^[22]^ We integrated TARGET-WT clinical data with GEO expression profiles, identifying 61 DEIRGs. LASSO and Cox regression analyses identified 4 prognostic DEIRGs (EGF, TDGF1, LTB4R, and HLA-DMB). We developed a prognostic risk score model. The model effectively stratifies patients into high-risk and low-risk groups with significantly different outcomes. Time-dependent ROC analysis demonstrated the model’s strong predictive accuracy for 1-, 3-, and 5-year OS (AUC = 0.691, 0.679, and 0.707, respectively), confirming its prognostic value in WT.

The TME plays a crucial role in WT initiation and progression.^[23]^ We conducted in-depth analysis of immune microenvironment differences between high-risk and low-risk subgroups. Significant differences in immune cell infiltration patterns were observed between subgroups, with increased infiltration of antitumor immune cells (including activated CD8+ T cells and regulatory T cells). These findings align with Mattis et al’s demonstration that stromal CD8+ T cell infiltration correlates with improved outcomes and prolonged progression-free survival (PFS) in WT.^[15]^ Investigating inflammation-immune microenvironment interactions could inform WT treatment strategies.^[24,25]^ COX-2-mediated inflammatory activation may suppress immune surveillance in WT, suggesting COX-2 as a potential therapeutic target.^[26]^ Immune microenvironment composition and function significantly influence WT prognosis. Low-risk patients may maintain tumor control through preserved immune function and cellular differentiation. High-risk patients likely experience accelerated progression from immune dysregulation.^[27]^

Tumor heterogeneity represents a key characteristic of WT, driven by dysregulated signaling pathways.^[28,29]^ GSVA revealed significant enrichment of genomic instability pathways (DNA replication, RNA metabolism, G2/M checkpoint dysregulation) in high-risk patients. Low-risk patients showed enrichment in immune regulation, metabolic reprogramming, and cell signaling pathways.^[30]^ Studies demonstrate that throughout the cell cycle and DNA replication, chromatin integrity is crucial for genome stability maintenance.^[31]^ Chromatin instability represents a fundamental driver of tumorigenesis.^[32]^ FBXO45 deficiency or downregulation leads to disrupted PPP6C-UPF1 interaction, causing persistent UPF1 hyperphosphorylation, resulting in histone depletion, chromatin relaxation, genomic instability, and elevated mutation rates, ultimately promoting LUAD development and tumor heterogeneity.^[33]^ Leuzzi et al demonstrated that SMARCAL1, a genome stability protein, reduces endogenous DNA damage during replication, maintaining genome stability while suppressing innate immunity and promoting immunosuppressive molecules to evade immune surveillance.^[34]^ These findings link signaling pathway dysregulation to tumor heterogeneity. High-risk patients exhibit greater tumor heterogeneity, likely from driver gene mutations (e.g., KRAS, PTEN).

Our drug sensitivity analysis offers potential guidance for personalized WT treatment. Low-risk patients exhibited heightened sensitivity to 26S proteasome inhibitors (bortezomib) and targeted therapies (dasatinib, lapatinib, and rapamycin). Lapatinib reversibly inhibits HER2 and epidermal growth factor receptor tyrosine kinase domains, blocking PI3K/AKT and mitogen-activated protein kinase (MAPK) signaling pathways, and is clinically approved for HER2+ breast cancer.^[35,36]^ Bortezomib selectively targets the 26S proteasome, preventing protein degradation in cancer cells, with clinical efficacy in multiple myeloma^[37]^ and lymphoma.^[38]^ This proteasome inhibition induces apoptosis. Collectively, these drugs represent potential therapeutic options for low-risk WT patients. Future studies should validate these drugs in clinical WT samples and incorporate drug sensitivity metrics to refine the prognostic model, thereby enhancing its clinical utility.

We systematically analyzed pan-cancer expression patterns of hub genes EGF and LTB4R, revealing their potential pan-cancer biological functions. EGF/epidermal growth factor receptor (EGFR) expression correlates significantly with prognosis across multiple cancers. Head and neck squamous cell carcinoma patients with below-median EGF expression showed poorer 5-year survival.^[39]^ This aligns with our observed positive EGF-immune activator correlation, likely reflecting EGF’s roles in proliferation and differentiation through PI3K/AKT and MAPK pathway activation that enhances immune cell function and promotes immune responses.^[40,41]^ Conversely, LTB4R’s negative correlation with immune activators suggests immunosuppressive functions. As an IRG, LTB4R significantly impacts prognosis in colorectal cancer^[42]^ and renal cell carcinoma.^[43]^ LTB4R critically regulates tumor development, progression, and metastasis.^[44]^ Zhang et al demonstrated LTB4R’s oncogenic role in renal cell carcinoma. LTB4R overexpression promotes proliferation, migration, invasion and suppresses apoptosis in renal carcinoma cells.^[45]^ through activation of PI3K/AKT and MAPK/ERK pathways, promoting tumor cell proliferation and survival.^[42]^ In summary, we identified EGF and LTB4R as novel prognostic biomarkers for WT, which demonstrate strong prognostic value and therapeutic potential in WT patients.

While this study advances prognostic modeling and immune microenvironment analysis in WT, several limitations should be noted. First, our findings rely solely on retrospective public database analyses without external cohort validation, potentially limiting model generalizability and stability. Multicenter prospective studies are needed for validation. Second, while EGF and LTB4R demonstrate oncogenic roles in other cancers, their molecular mechanisms in WT remain uncharacterized and require functional validation through in vitro and in vivo studies.

Despite these limitations, our study establishes a foundation for prognostic evaluation and personalized therapy in WT. Future research should focus on: refining the prognostic model, characterizing dynamic immune microenvironment changes, and integrating experimental validation with clinical translation. These efforts will advance precision medicine and personalized management strategies for WT.

## Acknowledgments

The authors thank the TCGA and GEO consortia for making data publicly available.

## Author contributions

**Conceptualization:** Jin Chen.

**Data curation:** Jin Chen, Guobin Yang, Jun Liao.

**Formal analysis:** Jin Chen.

**Methodology:** Jin Chen.

**Project administration:** Jin Chen.

**Resources:** Zhihui Zhu, Huajian Gu.

**Supervision:** Huajian Gu.

**Visualization:** Jin Chen.

**Writing – original draft:** Jin Chen.

**Writing – review & editing:** Huajian Gu.




