# Supplementary material for: Identification of immune-related prognostic genes and construction of a risk model for Wilms tumor: A retrospective bioinformatics study
Source: Medicine (Baltimore). 2026 Jul 24;105(30):e49868. doi: 10.1097/MD.0000000000049868 (PMC13406133; doi:10.1097/MD.0000000000049868)
Supplement: Supplementary file 1 [file medi-105-e49868-s001.pptx]

## Slide 1
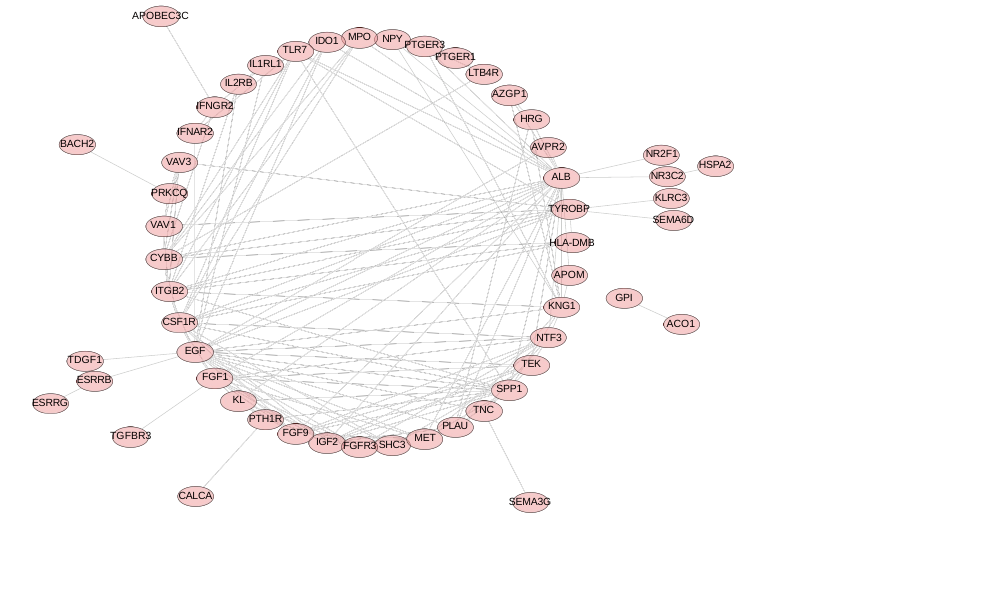

APOBEC3C
MPO
NPY
IDO1
PTGER3
TLR7
PTGER1
IL1RL1
LTB4R
IL2RB
AZGP1
IFNGR2
HRG
IFNAR2
BACH2
AVPR2
NR2F1
VAV3
HSPA2
NR3C2
ALB
PRKCQ
KLRC3
TYROBP
SEMA6D
VAV1
HLA-DMB
CYBB
APOM
ITGB2
GPI
KNG1
CSF1R
ACO1
NTF3
EGF
TDGF1
TEK
FGF1
ESRRB
SPP1
KL
ESRRG
TNC
PTH1R
PLAU
FGF9
TGFBR3
MET
IGF2
SHC3
FGFR3
CALCA
SEMA3G
