# Supplementary material for: Identification of immune-related prognostic genes and construction of a risk model for Wilms tumor: A retrospective bioinformatics study
Source: Medicine (Baltimore). 2026 Jul 24;105(30):e49868. doi: 10.1097/MD.0000000000049868 (PMC13406133; doi:10.1097/MD.0000000000049868)
Supplement: Supplementary file 2 [file medi-105-e49868-s002.pptx]

## Slide 1
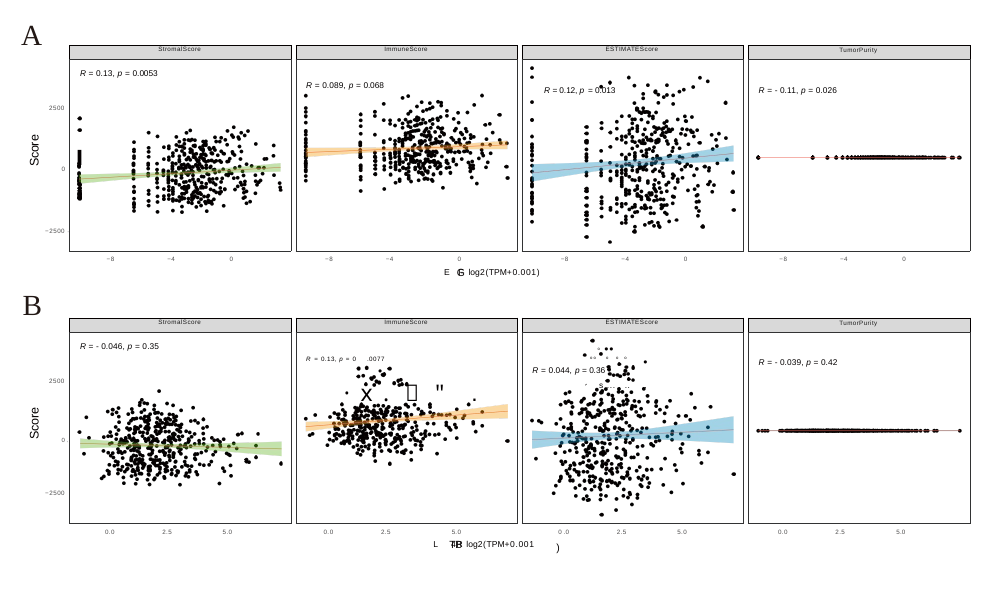

A
| StromalScore |
| --- |
| R = 0.13, p = 0.0053 |
| |
| |
| ImmuneScore |
| --- |
| R = 0.089, p = 0.068 |
| |
| |
| ESTIMATEScore |
| --- |
| R = 0.12, p = 0.013 |
| TumorPurity |
| --- |
| R = - 0.11, p = 0.026 |
2500
0
−2500 -
Score
	 E F log2(TPM+0.001)
B
−8 −4 0 −8 −4
0
−8 −4 0 −8 −4 0
G
| StromalScore |
| --- |
| R = - 0.046, p = 0.35 |
| |
| |
| ImmuneScore |
| --- |
| R = 0.13, p = 0 .0077 |
| |
| |
| ESTIMATEScore | | |
| --- | --- | --- |
| o ●● oo o o o R = 0.044, p = 0.36 、 s ... . .. . | | |
| | | |
| | | |
| TumorPurity |
| --- |
| R = - 0.039, p = 0.42 |
●
●
2500
0 -
−2500
·x.燃" .
Score
	 L 4R log2(TPM+0.001
0.0 2.5 5.0 0.0 2.5
5.0
0
.0 2.5 5.0 0.0 2.5 5.0
)
TB
